# E2F-1 induces melanoma cell apoptosis via PUMA up-regulation and Bax translocation

**DOI:** 10.1186/1471-2407-7-24

**Published:** 2007-01-30

**Authors:** Hongying Hao, Yanbin Dong, Maria T Bowling, Jorge G Gomez-Gutierrez, H Sam Zhou, Kelly M McMasters

**Affiliations:** 1 Department of Surgery, University of Louisville, Louisville, KY 40292, USA; 2 J. Graham Brown Cancer Center, Departments of Medicine, and Microbiology and Immunology, University of Louisville, Louisville, KY 40292, USA

## Abstract

**Background:**

PUMA is a pro-apoptotic Bcl-2 family member that has been shown to be involved in apoptosis in many cell types. We sought to ascertain whether induction of PUMA plays a crucial role in E2F-1-induced apoptosis in melanoma cells.

**Methods:**

PUMA gene and protein expression levels were detected by real-time PCR and Western blot in SK-MEL-2 and HCT116 cell lines after Ad-E2F-1 infection. Activation of the PUMA promoter by E2F-1 overexpression was detected by dual luciferase reporter assay. E2F-1-induced Bax translocation was shown by immunocytochemistry. The induction of caspase-9 activity was measured by caspase-9 colorimetric assay kit.

**Results:**

Up-regulation of the PUMA gene and protein by E2F-1 overexpression was detected by real-time PCR and Western blot analysis in the SK-MEL-2 melanoma cell line. In support of this finding, we found six putative E2F-1 binding sites within the PUMA promoter. Subsequent dual luciferase reporter assay showed that E2F-1 expression could increase the PUMA gene promoter activity 9.3 fold in SK-MEL-2 cells. The role of PUMA in E2F-1-induced apoptosis was further investigated in a PUMA knockout cell line. Cell viability assay showed that the HCT116 PUMA-/- cell line was more resistant to Ad-E2F-1-mediated cell death than the HCT116 PUMA+/+ cell line. Moreover, a 2.2-fold induction of the PUMA promoter was also noted in the HCT116 PUMA+/+ colon cancer cell line after Ad-E2F-1 infection. Overexpression of a truncated E2F-1 protein that lacks the transactivation domain failed to up-regulate PUMA promoter, suggesting that PUMA may be a transcriptional target of E2F-1. E2F-1-induced cancer cell apoptosis was accompanied by Bax translocation from the cytosol to mitochondria and the induction of caspase-9 activity, suggesting that E2F-1-induced apoptosis is mediated by PUMA through the cytochrome C/Apaf-1-dependent pathway.

**Conclusion:**

Our studies strongly demonstrated that E2F-1 induces melanoma cell apoptosis via PUMA up-regulation and Bax translocation. The signaling pathways provided here will further enhance insights on the mechanisms of E2F-1-induced cancer cell apoptosis as a strategy for cancer therapy.

## Background

E2F-1 is the best characterized member of the E2F family of transcription factors, which is comprised of six members that have the ability to regulate the cell cycle. The E2F family regulates overlapping sets of target genes, and all contain related DNA binding and dimerization domains. All members of the family except E2F-6 also contain a transactivation domain [1]. Overexpression of E2F-1 has demonstrated induction of S-phase entry as well as apoptosis [2]. Our previous studies have shown that infection of Ad-E2F-1, an adenovirus vector that expresses the full-length E2F-1 gene, can efficiently induce apoptosis in a wide spectrum of cancer cells *in vitro *as well as *in vivo *[3-5]. So far, E2F-1 has been implicated in the induction of apoptosis through p53-dependent and p53-independent pathways [6,7]. Several genes involved in the activation or execution of apoptosis recently have been identified as E2F-1 target genes, such as p14/p19ARF and p73 [6-10]. However, the molecular mechanisms underlying E2F-1-induced apoptosis are far from being fully understood.

Bcl-2 family members, which consist of both anti- and pro-apoptotic molecules, are major regulators of apoptosis [11]. PUMA (p53 up-regulated modulator of apoptosis) belongs to a subset of the Bcl-2 family – the pro-apoptotic BH3-only family. It shares only the amphipathic α-helical BH3 region with the rest of the Bcl-2 family members [12,13]. BH3-only members, including PUMA, Bid, Bad, Bim, and Noxa, serve to integrate diverse apoptotic stimuli into a common cell death pathway governed by other multi-domain Bcl-2 family members, such as Bax and Bak [14]. In healthy cells, Bax exists mainly in the cytosol in an inactive form. In response to apoptotic signals, Bax can be induced to undergo a conformational change and migrate from the cytosol to the mitochondria [15,16]. The conformationally altered Bax protein oligomerizes on the mitochondrial outer membrane, induces cytochrome *c *release, and then causes the cells to undergo apoptosis [17]. The antiapoptotic Bcl-2 family proteins, Bcl-2 or Bcl-xL, prevent the Bax conformational change by heterodimerizing with Bax, whereas various BH3-only proteins induce Bax conformational change in response to apoptotic stimuli [18-20].

The purpose of this study was to identify whether PUMA up-regulation and Bax translocation contributed to E2F-1-induced apoptosis in melanoma cells. Our data demonstrated that E2F-1 leads to up-regulation of PUMA at the transcription level in SK-MEL-2 cells. Sequence analysis showed that there were six putative E2F-1 binding sites within the PUMA promoter. Dual luciferase assay demonstrated that E2F-1 could activate PUMA promoter activity 9.3-fold. The HCT116 PUMA knockout cell line was resistant to E2F-1-mediated apoptosis in comparison with HCT116 cells containing the wild-type PUMA gene. Overexpression of a truncated E2F-1 protein that lacks the transactivation domain failed to up-regulate PUMA promoter, suggesting that PUMA may be an E2F-1 transcriptional target. Overexpression of E2F-1-induced apoptosis was also accompanied by Bax translocation from cytosol to mitochondria. Taken together, these results suggest that PUMA up-regulation and Bax translocation play an important role in E2F-1-induced melanoma cell apoptosis.

## Methods

### Cell lines and culture conditions

Human melanoma cell line SK-MEL-2 (with mutant p53) was purchased from ATCC and cultured in alpha-MEM medium supplemented with 10% heat-inactivated fetal bovine serum (FBS) and penicillin/streptomycin (100 units/mL). The human colon cancer cell line HCT116 PUMA-/- and control cell line HCT116 PUMA+/+ were generously provided by Dr. Bert Vogelstein [21] and were maintained in McCoy's 5A medium supplemented with 10% FBS and penicillin/streptomycin (100 units/mL). All cell culture reagents were obtained from Gibco/BRL (Bethesda, MD). Cells were cultured in a 5% CO2 incubator at 37°C, and the medium was changed every 3 days.

### Adenoviral vectors and infection conditions

Four replication-defective recombinant adenoviral vectors were used. The Ad5CMV-E2F-1 (Ad-E2F-1) vector has been deleted in the E1 subunit and contains the transgene E2F-1 under the control of the cytomegalovirus (CMV) promoter [3]. The Ad5CMV-LacZ (Ad-LacZ) was used as a control vector that expresses nuclear-localized β-galactosidase under control of the CMV promoter [3]. The Ad/T-E2FD vector expresses truncated E2F-1 protein, which retains E2F-1 DNA-binding, but lacks its transactivation domain. Expression of truncated E2F-1 from Ad/T-E2FD was controlled by a tetracycline inducible promoter. The Adeno-X Tet-off virus (AdTet-off) was obtained from Clontech (Mountain View, CA) and was used at multiplicity of infection (MOI) 5 as a helper virus to activate the tetracycline inducible promoter of Ad/T-E2FD. Where indicated, doxycycline (Sigma, St. Louis, MO) was added to a final concentration of 1 μg/mL. All vectors were propagated in the 293 cell line, and titers were determined using standard plaque assays [22].

The infection procedure used has been described in detail previously [3]. Cells were plated into 6-well plates at 2 × 10^5 ^cells/well or 24-well plates at 4 × 10^4 ^cells/well for SK-MEL-2 cells and 1 × 10^5 ^cells/well in 24-well plate for HCT116 cells. The following day, the media were removed, and the adenoviral vectors were added in 200 μL of medium without FBS for 6-well plates or 100 μL of medium for 24-well plates at indicated multiplicities of infection (MOIs). Mock infection was performed by treatment of cells with vehicle (media) only. After 2 hours of incubation at 37°C, fresh alpha-MEM with 5% FBS (for SK-MEL-2 cells) or McCoy's 5A with 5% FBS (for HCT116 cells) was added to bring the final volume up to 2 mL for 6-well plates and 1 mL for 24-well plates. Cells were then cultured at 37°C and harvested at selected time points for analysis.

### Luciferase-promoter assay

Cells were plated in a 12-well plate at 1 × 10^5 ^cells/well for SK-MEL-2 cells or at 2.2 × 10^5 ^cells/well for HCT116 PUMA+/+ cells and cultured in medium containing 10% FBS. Within 24 hours, cells were co-transfected with a PUMA promoter construct and a phRL-CMV plasmid (containing the cDNA encoding Renilla luciferase) by using Lipofectamine 2000 transfection reagent (Invitrogen, Carlsbad, CA). Cells were infected with Ad-LacZ or Ad-E2F-1 6 hours later. After 16 hours of infection, lysates were collected and assayed for luciferase activity by using the Dual Luciferase Reporter Assay System (Progema, Madison, WI). Luciferase activity from untreated control cells was used for the background signal. All of the luciferase values were normalized to the Renilla luciferase readout values and expressed as x-fold induction relative to that of Ad-LacZ infected cells.

The PUMA promoter (pBV-Luc) reporter construct has been previously described [13] and was a kind gift from Dr. Bert Vogelstein (The Howard Hughes Medical Institute, Baltimore, MD). The phRL-CMV plasmid was a kind gift from Dr. Sham S. Kakar (University of Louisville, Louisville, KY).

### Real-time PCR analysis and Western blot analysis

Real-time PCR was performed using the SYBR Green PCR Master Mix kit, according to the protocol of the manufacturer [ABI, Foster City, CA] Briefly, melanoma cells or colon cancer cells were infected with Ad-E2F-1 or Ad-LacZ at MOI 100. At indicated time points after infection, total RNA was isolated using RNeasy mini kit, along with RNase Free DNase set to remove any traces of DNA contamination (QIAGEN, Valencia, CA). cDNA was prepared from 500 ng of total RNA with TaqMan Reverse Transcription Reagents (ABI/Roche, Branchburg, NJ). Real-time PCR was performed using 12.5 ng of cDNA as a template for each gene. Reactions were carried out in a 96-well Optical Reaction plate (ABI) using the ABI Prism 7000 sequence detection system. Primers for the PUMA gene have been described previously [23]. Data were analyzed using the comparative C_T _method [24] and were presented as mean ± standard deviation (SD) from two independent experiments. Western blot analysis was performed as described previously [4]. Human PUMA, E2F-1, and anti-actin were detected using the antibodies ab9643-100 (1: 500, Abcam Ltd, Cambridge, UK), sc-251 (1:1000, Santa Cruz, California, CA), and A-2066 (1: 5000, Sigma-Aldrich, St. Louis, MO). Protein bands on film from Western blots were scanned, and the optical density of the bands was quantified using the NIH image 1.61 software.

### Cytotoxicity and cell viability assay

Cytotoxicity of Ad-E2F-1 infection in HCT116 PUMA+/+ and HCT116 PUMA-/- cells was assessed by MTT assay. Briefly, early passage PUMA-/- and PUMA+/+ cells were cultured in a 24-well plate at 1 × 10^5 ^cells/well in 1 mL medium. The next day, cells were infected at the indicated MOI of Ad-E2F-1 or Ad-LacZ. Three days later, 10% of the original volume (100 μL) of MTT (5 mg/mL MTT in PBS, Sigma, St. Louis, MO) was added to each well and incubated at 37°C for 4 hours. After incubation, 500 μL of lysis buffer (10%SDS in 0.01N HCl) was added, and samples were incubated at 37°C overnight for complete cell lysis. On the following day, cytotoxicity was assessed by measuring the conversion of the tetrazolium salt, MTT, to formazan through measurement of absorbance at 570 nm. The results are expressed as the percentage of absorbance of the control (untreated) cells from triplicate samples.

For cell viability assay, cells were plated at 7 × 10^5 ^cells/dish in 60 mm dishes and treated as above. Cell viability was determined at indicated time points using the trypan blue exclusion method. Cells were stained with trypan blue (final concentration, 0.2%) for 5 minutes and counted using a hemacytometer.

### Immunocytochemistry

SK-MEL-2 cells, HCT116 PUMA+/+, and HCT116 PUMA-/- cells were seeded on cover slips in 12-well plates and cultured in a 5% CO_2 _incubator at 37°C. The following day, cells were infected with Ad-E2F-1 or Ad-LacZ at MOI 100. Mock infection was performed by treatment of cells with vehicle (media) only. After infection 24 hours, the media was changed to fresh culture medium and cells were incubated with MitoTracker Red 580 (Invitrogen Corporation, Carlsbad, CA) with the final concentration of 300 nM in 37°C for 40 minutes. The cover slips were rinsed with warm medium twice and fixed with 3.7% formaldehyde in 37°C for 15 minutes. The cells were then permeabilized with ice cold acetone for 5 minutes. Blocking was performed in PBS supplemented with 5% goat serum. Primary antibody Bax (Santa Cruz Biotechnology, Inc., Santa Cruz, CA) was applied at 1:100 at room temperature for 45 minutes. Then, the cells were washed three times for 30 minutes and incubated with the secondary antibody (goat anti-mouse Alex 488 at 1:500) (Invitrogen Corporation, Carlsbad, CA) at room temperature for 40 minutes. Cells were counterstained with 4',6-diamidino-2-phenylindole (DAPI) (Santa Cruz Biotechnology, Inc., Santa Cruz, CA) at 200 nM for 2 minutes in room temperature. Finally, cells were washed extensively and mounted with Mowiol (Calbiochem, La Jolla, CA). The scans were done in a sequential mode to avoid channel crosstalk. Pictures were taken on an Olympus Fluoview 500 confocal microscope.

### Determination of caspase-9 activity

The caspase-9 colorimetric assay kit (from R&D Systems, Minneapolis, MN) was used for caspase-9 activity assay. Briefly, cells were seeded in a 100-mm dish at 1 × 10^6 ^cells/dish for melanoma cells or 2 × 10^6 ^cells/dish for HCT116 cells. The next day, cells were infected at the indicated MOI of Ad-E2F-1 or Ad-LacZ. The 2 × 10^6 ^cells were harvested and pelleted at indicated times. Cells were resuspended in 50 μL of chilled lysis buffer, incubated on ice for 10 minutes, and centrifuged for 1 minute in a microcentrifuge (10,000 g). Supernatants (cytosolic extract) were transferred to a fresh tube and put on ice to assay protein concentration. Protein concentration was adjusted with the cell lysis buffer. Then, 50 μL of the cell lysate was added to 50 μL of 2× reaction buffer and 5 μL of caspase-9 substrate (LEHD-pNA) and incubated at 37°C for 1 hour to test for protease activity by the addition of a caspase-specific peptide that is conjugated to the color reporter molecule p-nitroanaline (pNA). The cleavage of the peptide by the caspase releases the chromophore pNA, which can be quantitated at 405 nm. The level of caspase activity in the cell lysate is directly proportional to the color reaction. No cell lysates and no substrates were used as blanks. The results were expressed as the fold increase in Ad-E2F-1-treated cells over that of Ad-LacZ-treated cells.

## Results

### E2F-1 leads to increased PUMA expression in SK-MEL-2 cells

**Figure 1:**
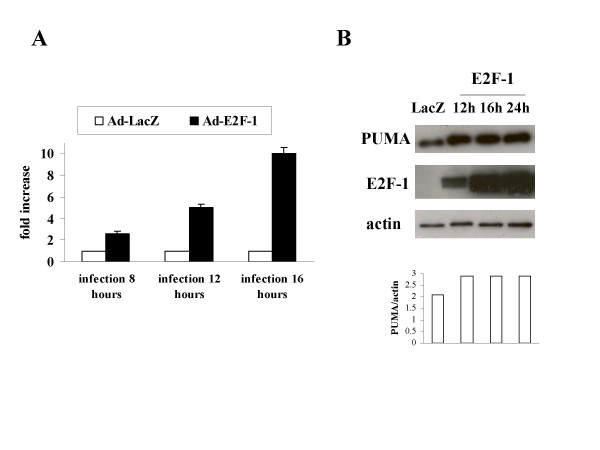
**A. Up-regulation of PUMA at the transcription level after Ad-E2F-1 expression in SK-MEL-2 cells. **SK-MEL-2 cells were infected with Ad-E2F-1 at MOI 100. Ad-LacZ was used as control. Cells were harvested after infection at 8 hours, 12 hours, and 16 hours. Real-time PCR was performed as described in Materials and Methods. β-actin was used as an internal control. The bar graphs are expressed as fold increase relative to Ad-LacZ-infected cells adjusted for β-actin. Each of the experiments is a representation of two independent experiments performed in duplicate. After adjustment for β-actin mRNA expression, real-time PCR results showed a 2.6-, 5-, and 10-fold induction at the PUMA mRNA level after 8 hours, 12 hours and 16 hours of Ad-E2F-1 infection, respectively. **B. Up-regulation of PUMA at the protein level after Ad-E2F-1 expression in SK-MEL-2 cells. **Western blot analysis of PUMA at indicated time points after Ad-LacZ or Ad-E2F-1 infection. Anti-actin was used to show the equal loading and transfer in each lane. E2F-1 expression was also shown at the indicated time points to correlate with PUMA expression. PUMA/actin showed the ratio of the scanned optical density of each protein band. In SK-MEL-2 cells, PUMA protein level increased at 8 hours post-infection with Ad-E2F-1 and was still pronounced at 16 hours and 18 hours post-infection with Ad-E2F-1.

Our previous studies have shown that adenovirus-mediated overexpression of E2F-1 can efficiently induce apoptosis in a wide spectrum of cancer cell lines. Due to the importance of the Bcl-2 family members in the control of cellular fate (survival versus apoptosis), we focused our present study on determining whether PUMA, one of the BH3 only family members, was involved in E2F-1-induced cell apoptosis. We chose SK-MEL-2 melanoma cell line because all of the other melanoma cell lines typically express p53, whereas only this cell line has mutant p53, and there is no p73 expression after Ad-E2F-1 infection (unpublished data). We examined the effect of E2F-1 expression on PUMA mRNA expression over time. Quantitative real-time PCR analysis showed that a 2.6-, 5-, and 10-fold increase of PUMA mRNA levels was observed in SK-MEL-2 cells after E2F-1 infection at 8 hours, 12 hours, and 16 hours, respectively, compared to the Ad-LacZ control (Fig. 1A).

To validate the PUMA mRNA up-regulation findings after E2F-1 treatment, we performed Western blotting to analyze the PUMA gene expression at the protein level. As shown in Fig. 1B, the PUMA protein level increased at 8 hours and was still pronounced at 16 hours and 18 hours post-infection with Ad-E2F-1. The alterations of the PUMA protein level corresponded with changes in the mRNA level. These data suggest that induction of PUMA may be an important event in E2F-1-induced cell apoptosis.

### Identification of a putative E2F-1 binding site in the human PUMA promoter

The fact that overexpression of E2F-1 induces expression of PUMA suggests that the PUMA gene may be one of the potential targets of E2F-1. The presence of E2F-1 responsive elements in the PUMA promoter would be a strong indication that E2F-1 regulates PUMA at the transcription level. Thus, we performed sequence analysis to determine whether any putative E2F-1 binding sites existed in the PUMA gene promoter. A previous study showed that E2F-1 interacts with all of the G-residues in the sequence GGCGGGAAA, while the A-residues are not required for DNA binding [25]. Six potential E2F-1 binding sites (GGCGGG) were found at -63/-68, -73/-78, -79/-84, -100/-105, -124/-129, and -132/-137 basic regions of the PUMA promoter (the position is relative to the ATG initiation codon). This further suggests that PUMA might be a transcriptional target of E2F-1.

### Ad-E2F-1 infection up-regulates the activity of the PUMA promoter

**Figure 2:**
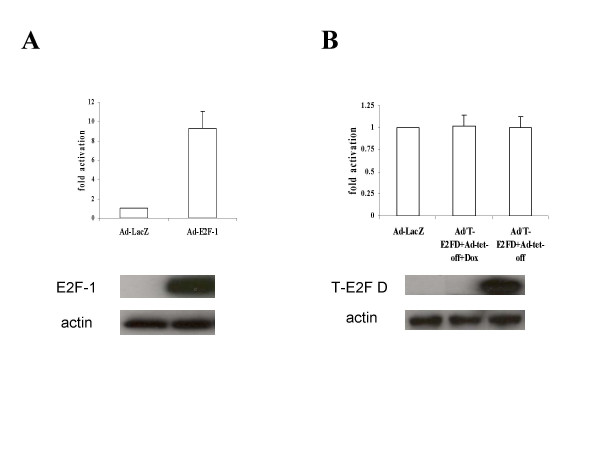
**A. E2F-1 expression up-regulates the activity of the PUMA promoter in SK-MEL-2 cells**. SK-MEL-2 cells were co-transfected with a total of 0.88 μg of DNA, including 0.8 μg of PUMA promoter reporter plasmid and 0.08 μg phRL-CMV plasmid (as an internal control) in a 12-well plate by using Lipofectamine 2000 transfection reagent. After 6 hours of transfection, the cells were infected with Ad-LacZ (as control) and Ad-E2F-1. Luciferase activity was examined 16 hours later as described in the manufacturer's protocol using the Dual Luciferase Reporter Assay System (Promega). The bar graphs depict the fold of activation in the luciferase assay after normalization for Renilla luciferase readout values. Each of the experiments is a representation of at least three independent experiments performed in duplicate. **B. Transactivation domain of E2F-1 was required for the up-regulation of the PUMA promoter in SK-MEL-2 cells. **SK-MEL-2 cells were co-transfected as in Fig. 2A. After 6 hours of transfection, the cells were infected with Ad/T-E2FD (expressing truncated E2F-1) at MOI 100 plus AdTet-off at MOI 5 (using as a helper virus), Ad/T-E2FD at MOI 100 plus AdTet-off at MOI 5, and doxycycline 1 μg/ml or Ad-LacZ at MOI 100. Luciferase activity was examined 16 hours later as in Fig. 2A. The bar graphs depict the fold of activation in the luciferase assay after normalization for Renilla luciferase readout values. Each of the experiments is a representation of at least three independent experiments performed in duplicate.

Although the above data suggests that E2F-1 transcriptionally up-regulates the PUMA gene, the additional test is to determine whether E2F-1 overexpression can transactivate a chimeric construct in the PUMA promoter linked to a reporter gene. For these experiments, we co-transfected SK-MEL-2 cells with a PUMA promoter reporter plasmid (PUMA promoter linked to a luciferase reporter gene, and the PUMA promoter fragment was -428/+67 region of the human PUMA promoter) and a phRL-CMV plasmid (containing the cDNA encoding Renilla luciferase, used as an internal control for transfection efficiency). After 6 hours of transfection, the cells were infected with Ad-LacZ (as control) and Ad-E2F-1. Luciferase activity was examined after 16 hours of infection. Ad-E2F-1 infection increased PUMA promoter activity by 9.3 fold in SK-MEL-2 cells compared with the control cells infected with Ad-LacZ (Fig. 2A). This result suggests that Ad-E2F-1 infection can activate the PUMA promoter in SK-MEL-2 cells. This also provides additional evidence that PUMA might be a transcriptional target of E2F-1.

### E2F-1 leads to increased PUMA expression is dependent on its transactivation domain in SK-MEL-2 cells

To confirm that the transactivation function of E2F-1 is required for the up-regulation of PUMA in SK-MEL-2 cells, we performed PUMA promoter luciferase assay after infection with Ad/T-E2FD (an adenovirus vector expressing truncated E2F-1 protein without its transactivation domain) at MOI 100. Ad/T-E2FD infection did not increase PUMA promoter activity in SK-MEL-2 cells compared with the control cells infected with Ad-LacZ (Fig. 2B). These data indicate that the transactivation domain of E2F-1 is required for the increase of PUMA in SK-MEL-2 cells.

### Loss of PUMA protects the HCT116 cells against E2F-1-induced cell death

**Figure 3:**
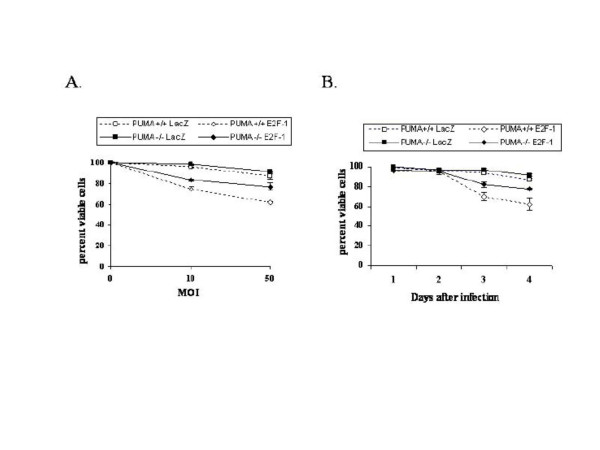
**Resistance in response to E2F-1 expression in the HCT116 PUMA-/- cell line compared to the HCT116 PUMA+/+ cell line by cell viability assay. **Both floating and adherent cells were harvested, counted, and scored at indicated time points. Viability was assessed using the trypan blue exclusion method. Results are expressed as the percent viable cells at each point and values represent the means ± SD (bars) of three independent experiments. **A. **HCT116 PUMA-/- and HCT116 PUMA+/+ cell lines were treated by infection with Ad-LacZ or Ad-E2F-1 at indicated MOI. The viability was assessed at day 4 after virus infection. **B. **Time course of cell viability of HCT116 PUMA-/- and HCT116 PUMA+/+ cells in response to Ad-LacZ or Ad-E2F-1 infection at MOI of 50.

To further evaluate the importance of the PUMA gene in Ad-E2F-1-induced apoptosis, we took advantage of a PUMA knockout cell line. We reasoned that if PUMA is truly a mediator in E2F-1-induced apoptosis, there should be significant resistance to E2F-1-induced cell death in PUMA knockout cells. The HCT116 PUMA knockout cell line (PUMA-/-) and the HCT116 control cell line (PUMA+/+) were infected with Ad-E2F-1 at the indicated MOI and indicated times and were then subjected to analysis of cell viability and a cytotoxicity assay. As shown in Fig. 3A, HCT116 PUMA-/- cells were more resistant to E2F-1-induced cell death, while HCT116 PUMA+/+ cells were more sensitive to E2F-1-induced cell death, especially at a higher MOI (MOI 50). After 4 days of Ad-E2F-1 infection, analysis of HCT116 PUMA-/- cells revealed 15% more viable cells than PUMA+/+ cells (Fig. 3B). MTT assay also showed that loss of the PUMA gene enhanced survival of HCT116 cells infected with Ad-E2F-1 compared with Ad-LacZ infection. After 4 days of Ad-E2F-1 infection at MOI 50, analysis of HCT116 PUMA-/- cells revealed 18% more viable cells than PUMA+/+ cells by MTT test (data not shown). To exclude the possibility that the difference in cell viability is due to different transduction efficiency of adenoviral vectors, we performed a β-galactosidase assay [3]. HCT116 PUMA+/+ and HCT116 PUMA-/- cell lines were infected with Ad-LacZ at MOI of 100. Transduction efficiency was determined by measuring the blue cells after cytochemical staining with X-gal 24 hours after infection. Equivalent transduction efficiency (approximately 70%) was observed in both cell lines (data not shown). These data suggest that loss of PUMA protects cells against E2F-1-mediated cell death, and up-regulation of PUMA plays an important role in E2F-1-induced cell death.

### Overexpression of E2F-1 increases the endogenous PUMA expression in HCT116 cells

**Figure 4:**
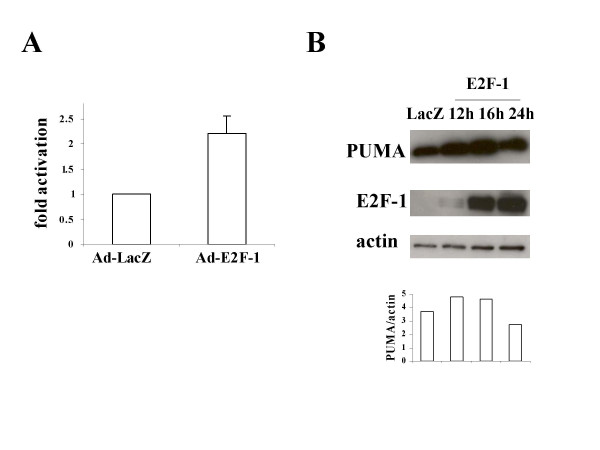
**A. E2F-1 expression up-regulates the activity of the PUMA promoter in HCT116 PUMA+/+ cells**. HCT116 PUMA+/+ cells were co-transfected with a total of 0.82 μg of DNA, including 0.8 μg of PUMA promoter reporter plasmid and 0.02 μg phRL-CMV plasmid (as an internal control) in a 12-well plate by using Lipofectamine 2000 transfection reagent, and then processed as in Fig. 2A. **4B Up-regulation of PUMA at the protein level after Ad-E2F-1 expression in HCT116 PUMA+/+ cells. **Western blot analysis of PUMA was performed in HCT116 PUMA+/+ cells at indicated time points as in Fig. 1B. PUMA/actin showed the ratio of the scanned optical density of each protein band. PUMA protein level was increased at 12 hours and 16 hours of infection with Ad-E2F-1.

To confirm the involvement of the PUMA gene in E2F-1-induced apoptosis in the HCT116 PUMA+/+ cell line, we again performed promoter assay, real-time PCR, and Western blot analysis in this cell line after treatment with Ad-E2F-1. As expected, the PUMA promoter was activated 2.2-fold after Ad-E2F-1 infection in HCT116 PUMA+/+ cells (Fig. 4A). Similarly, real-time PCR showed a 2-fold increase in PUMA expression in HCT116 PUMA+/+ cells at 6 hours after Ad-E2F-1 infection compared to cells infected with the Ad-LacZ control (data not shown). Western blotting results still showed that the PUMA protein level was increased at 12 hours and 16 hours of infection with Ad-E2F-1. The increased PUMA protein expression corresponded with the up-regulation of PUMA mRNA after Ad-E2F-1 infection in this cell line (Fig. 4B).

### Overexpression of E2F-1 is associated with Bax activation and translocation to mitochondria

**Figure 5:**
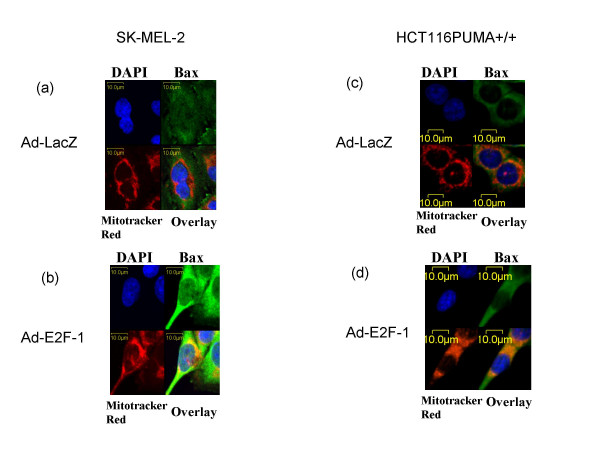
**Bax mitochondrial translocation after Ad-E2F-1 expression in SK-MEL-2 cells and HCT116 PUMA+/+ cells. **SK-MEL-2 cells **(a,b) **and HCT116PUMA+/+ cells **(c,d) **were infected with Ad-LacZ and Ad-E2F-1. After 24 hours of infection, cells were stained with Mitotracker Red 580 and Bax (green), then counterstained with DAPI (blue) as described in the Methods. After 24 hours of Ad-E2F-1 infection in SK-MEL-2 cells **(b) **and HCT116PUMA+/+ cells **(d)**, Bax clustered and translocated to mitochondria (an overlay of the three stains is given in yellow, which shows co-localization of Bax in mitochondria).

Recent evidence implicates Bax to be an important mediator of PUMA-activated apoptotic signals. To examine the possibility of PUMA eliciting Bax mitochondrial translocation when PUMA is transcriptionally up-regulated by E2F-1, we performed immunocytochemistry to study the cellular distribution of Bax with confocal microscope. Confocal microscopy revealed that Bax was diffusely distributed throughout the cytosol after 24 hours of Ad-LacZ infection or mock infection in SK-MEL-2 cells (Fig. 5a). After 12 hours of Ad-E2F-1 infection, Bax has the similar cytosol distribution pattern as that in mock or Ad-LacZ-infected cells (data not shown). However, after 24 hours of Ad-E2F-1 infection, Bax clustered and co-localized with mitochondria (Fig. 5b). An overlay of the three stains is given in yellow, which shows co-localization of Bax in mitochondria. Similarly, in HCT116 PUMA+/+ cells, we also observed that Bax was diffusely distributed throughout the cytosol after 24 hours of Ad-LacZ or mock infection (Fig. 5c). Bax clustered and co-localized with the mitochondria after 24 hours of Ad-E2F-1 infection (Fig. 5d). Thus, our results suggest that PUMA up-regulation and subsequently Bax translocation appear to contribute to E2F-1-induced apoptotic pathway.

### Activation of caspase-9 accompanied with E2F-1-induced PUMA overexpression and apoptosis

**Figure 6:**
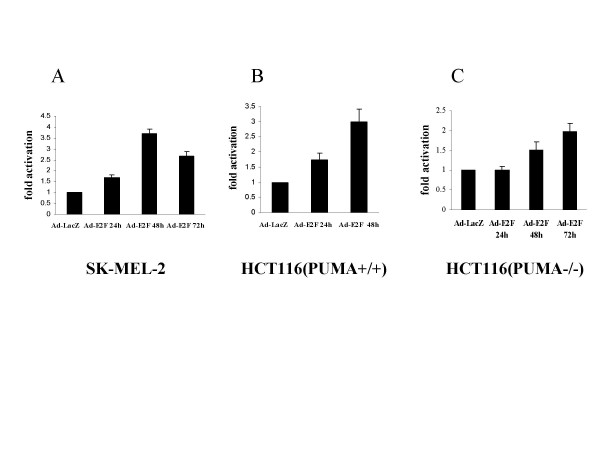
**Activation of caspase-9 was accompanied by PUMA-mediated E2F-1-induced apoptosis. **2 × 10^6 ^of SK-MEL-2 **(A), **HCT116 PUMA+/+ cells **(B)**, and HCT116PUMA-/- cells **(C) **were infected with Ad-E2F-1 or Ad-LacZ and lysed at indicated time points to collect their intracellular contents. The cell lysates were assayed for caspase-9 activity by measuring the digestion of p-nitroanaline (pNA) as a substrate. The OD405 values were measured, and the percentage values were compared with Ad-LacZ infected cells as indicated. Caspase-9 activity was induced 3.6 fold, 3.0 fold, and 1.5 fold after 48 hours of Ad-E2F-1 infection in SK-MEL-2 cells, HCT116 PUMA+/+ cells, and HCT116PUMA-/- cells, respectively.

Since PUMA up-regulation and Bax translocation have been shown to release cytochrome C from the mitochondria and activate caspase-9 through mitochondrial pathway [12,18], we performed caspase-9 activity assay to evaluate downstream effects of PUMA and Bax in E2F-1-induced apoptosis. SK-MEL-2, HCT116 PUMA+/+, and HCT116 PUMA-/- cells were infected with Ad-E2F-1 and lysed at indicated time points to collect intracellular contents. As shown in Figure 6, caspase-9 activity was induced after Ad-E2F-1 treatment in SK-MEL-2 and HCT116 PUMA+/+ cell lines, but not in control HCT116 PUMA+/+ cells infected with Ad-LacZ. In the SK-MEL-2 cell line, caspase-9 activity was induced 3.7-fold after 48 hours of Ad-E2F-1 infection. In the HCT116 PUMA+/+ cell line, caspase-9 activity was induced 3.0 fold after 48 hours of Ad-E2F-1 infection. In HCT116 PUMA-/- cells, caspase-9 activity was also increased after Ad-E2F-1 infection, but to a lesser degree (1.5 fold after 48 hours of Ad-E2F-1 infection). These data suggest that PUMA likely plays a role in mediating Ad-E2F-1-induced apoptosis through the cytochrome C/Apaf-1-dependent pathway.

## Discussion

To date, three pathways of E2F-1-induced apoptosis have been demonstrated: ***(1) ***A p53-dependent pathway through the stabilization of p53 by E2F-1; ***(2) ***p53-independent pathways through direct up-regulation of apoptotic related genes by E2F-1, such as p73 and PKR [26,27]; and ***(3) ***blocking of anti-apoptotic pathways through down-regulation or inhibition of anti-apoptotic molecules such as Bcl-2 and Mcl-1 [8]. Recently, a rapidly growing number of pro-apoptotic genes have been shown to be regulated by E2F-1 expression. Hershko et al [28] have demonstrated that E2F-1 up-regulates the expression of the pro-apoptotic BH3-only proteins, PUMA, Noxa, Bim, and Hrk/DP5, through a direct transcriptional mechanism in NIH3T3 cells. Another study, which screened a siRNA-expression library, identified one of the novel pathways involved in thapsigargin-induced apoptosis in HCT116 cells, in which endogenous E2F-1 might regulate TG-induced cell apoptosis via regulation of the expression of PUMA [29]. Loss of the PUMA gene in cancer cells has been shown to cause cells to be resistant to a variety of apoptotic stimuli such as DNA damage, microtubule-damaging agents [30], and ER stressor thapsigargin-induced apoptosis [31]. However, whether PUMA contributes to E2F-1-induced cancer cell apoptosis has not been fully elucidated. Our studies indicate that E2F-1 overexpression results in up-regulation of the pro-apoptotic PUMA gene at the transcription and protein levels. Sequence analysis shows that the human PUMA promoter contains six E2F-1 binding sites. Promoter assay demonstrates that E2F-1 can activate the PUMA promoter 9.3 fold in SK-MEL-2 cells. Our data also suggest that HCT116 PUMA knockout cells (PUMA-/-) are more resistant in response to E2F-1-induced apoptosis than wild-type PUMA cells (PUMA+/+). Activation of the PUMA promoter upon E2F-1 expression was observed by using Luciferase reporter assay in the HCT116 wild-type PUMA cell line (PUMA+/+). Furthermore, when we performed real-time PCR and Western blot analysis, the PUMA gene was up-regulated at both the transcriptional level and the protein level in this cell line. Collectively, all these data support the conclusion that PUMA is an important mediator of E2F-1-induced cancer cell apoptosis.

The E2F-1 protein contains a related DNA binding domain, dimerization domain, and a transactivation domain. Previous studies showed that the transactivation domain of E2F-1 is not required for E2F-1-induced apoptosis. In order to investigate the different apoptotic mechanisms of the full-length E2F-1 and E2F-1 without the transactivation domain, we constructed a new adenovirus vector, Ad/T-E2FD, which retains E2F-1 DNA-binding domain, but lacks its transactivation domain. Overexpression of a truncated E2F-1 protein lacking its transactivation domain failed to stimulate transcription from the PUMA promoter, suggesting that the transactivation domain is required for PUMA up-regulation in E2F-1-induced melanoma cell apoptosis.

PUMA was first identified as a novel gene by screening for p53-inducible target genes [12,13]. Studies with knockout mice or human cancer cells demonstrate that PUMA is essential for p53-dependent apoptosis induced by DNA damage [21,32,33]. Recently, Chipuk et al [34] report that PUMA provides a critical link that couples p53 transcription-dependent and transcription-independent functions. Genetic studies demonstrate the importance of PUMA for p53-mediated cell death and reinforce the control of PUMA to modulate the p53 response [35,36]. However, other studies showed that the activity of the PUMA promoter could be up-regulated by E2F-1 in a p53-independent manner [28]. Involvement of p53 in the E2F-1-induced increase of PUMA was also excluded by the finding that ectopic expression of E2F-1 in mouse primary fibroblasts derived from p53-null mice resulted in elevated PUMA levels [28]. Another study showed that PUMA mRNA was induced by p53-independent apoptotic stimuli [37]. In this study, we showed that PUMA promoter is activated by E2F-1 in SK-MEL-2 cells which express mutant p53. These data suggest that up-regulation of PUMA by E2F-1 overexpression in melanoma cells may not require functional p53.

Apoptosis can be initiated by a wide array of stimuli, including multiple signaling pathways that, for the most part, converge at the mitochondria. Several Bcl-2 family members, including PUMA and Bax, contribute to and regulate the apoptosis. The death effector, Bax, plays an essential role in the mitochondrial pathway of apoptosis. In the absence of death signals, Bax exists in the cytosol in an inactive form, but it becomes activated by a conformational change and is translocated to mitochondria during apoptosis. It has been reported that proapoptotic BH3-only protein, PUMA, promotes Bax mitochondrial translocation [38]. Here, our data showed that PUMA up-regulation after E2F-1 overexpression was subsequently accompanied by Bax mitochondrial translocation. Both PUMA and Bax function by releasing cytochrome C from mitochondria. The most important consequence of cytochrome C discharge is the activation of procaspase-9. This is facilitated by Apaf-1 and cytochrome C in the presence of dATP or ATP. Activated caspase-9 then triggers the processing and activation of the downstream caspase-3 and capase-7 that culminate in apoptotic cell death [39]**. **Here, we show that overexpression of E2F-1 causes induction of PUMA, mitochondrial translocation of Bax, which consequently leads to activation of caspase-9 and eventual apoptosis.

In this study, we noticed that HCT116 PUMA+/+ cells were more sensitive to E2F-1-induced cell death than HCT116 PUMA-/- cells. While loss of PUMA protected the HCT116 cells against E2F-1-induced cell death, the HCT116 PUMA-/- cells eventually underwent cell death after Ad-E2F-1 infection. Furthermore, in HCT116 PUMA-/- cells, caspase-9 activity was also increased after Ad-E2F-1 infection, albeit at a much lower level than SK-MEL-2 and HCT116 PUMA+/+ cells. Obviously, we cannot exclude other apoptotic pathways other than PUMA involved in Ad-E2F-1-induced cell apoptosis, such as p14/p19ARF, PKR, and other anti-apoptotic molecules such as Bcl-2 and Mcl-1. The results we observed in SK-MEL-2 melanoma cells may not be generalizable to all cancer cell types.

## Conclusion

In conclusion, our results suggest that induction of the pro-apoptotic molecule PUMA and subsequent Bax activation contributes to E2F-1-induced apoptosis in melanoma cells. Identification of specific genes in cancer cell apoptotic pathways may lead to the development of new cancer therapeutic strategies. The signaling pathways provided here further enhance our understanding of the mechanisms of E2F-1-induced cancer cell apoptosis, which has the potential for future clinical applications in cancer therapy.

## Abbreviations

FBS, fetal bovine serum; MOI, multiplicity of infection; SD, standard deviation; pNA, p-nitroanaline; DAPI, 4',6-diamidino-2-phenylindole.

## Competing interests

The author(s) declare that they have no competing interests.

## Authors' contributions

HH: carried out real-time PCR, Western blot, promoter assay, immunocytochemistry and drafted the manuscript.

YD and HSZ: participated in the design of the study.

MTB and JGG: helped to carry out Western blot.

KMM: designed the study and prepared the manuscript.

All authors read and approved the manuscript.

## Pre-publication history

The pre-publication history for this paper can be accessed here:
